# ﻿Two new species of *Pestalotiopsis* (Amphisphaeriales, Pestalotiopsidaceae) causing needle blight of *Pinus massoniana* in China

**DOI:** 10.3897/mycokeys.125.168320

**Published:** 2025-11-17

**Authors:** Hui Li, Yu-Qing Bai, Jun-Ya Xie, De-Wei Li, Li-Hua Zhu

**Affiliations:** 1 College of Forestry and Grassland, Nanjing Forestry University, Nanjing, Jiangsu 210037, China; 2 Co-Innovation Center for Sustainable Forestry in Southern China, Nanjing Forestry University, Nanjing, Jiangsu 210037, China; 3 The Connecticut Agricultural Experiment Station Valley Laboratory, Windsor, CT 06095, USA

**Keywords:** Asexual fungi, multi-locus phylogeny, new species, *
Pestalotiopsis
*, pine

## Abstract

*Pinus massoniana* Lamb. is an important tree species widely used for afforestation and industrial timber on barren hills in China. Needle blight of *P. massoniana*, caused by *Pestalotiopsis* spp., is widespread and occurs over a large area. In this study, 10 representative strains were isolated from diseased needles of *P. massoniana* in Anhui and Guangxi provinces. Based on phylogenetic analysis of three genomic loci (ITS, *TEF1*, and *TUB2*), combined with morphological characteristics, two new species—*Pestalotiopsis liuzhouensis***sp. nov.** and *Pestalotiopsis kendrickii***sp. nov.**—were identified. Pathogenicity experiments showed that these 10 representative strains were pathogenic to *P. massoniana*. This study enhances understanding of the diversity of pathogens causing *P. massoniana* needle blight and provides insights for future control strategies.

## ﻿Introduction

*Pinus massoniana* Lamb., as a unique native tree species in China, is widely distributed in the subtropical region (Lu et al. 2022). Adapted to arid and barren soil, *P. massoniana* needles and roots can be used in traditional Chinese medicine (He et al. 2009); its trunk can be used for papermaking and industrial construction (Liu et al. 2015); and it has a strong regeneration ability that can promote carbon cycling (Kang et al. 2006). Therefore, it has extremely high economic and ecological value (Yang et al. 2020). Needle blight was found in Quanjiao County, Anhui Province, and Liuzhou City, Guangxi Province, which seriously endangered the health of *P. massoniana* needles and must be monitored.

Pine wood nematode disease, caused by *Bursaphelenchus xylophilus* (pinewood nematode; PWN), is one of the main diseases affecting *P. massoniana* (Wu et al. 2022). As early as 1975, the Guizhou Forestry Institute investigated local diseases of *P. massoniana* forests and found diseases such as red blight, pine blister rust, pine leaf rust, and sooty blotch. Pine rot, a world-famous disease of young *P. massoniana*, is caused by *Cenangium ferruginosum* Fr. (Fu 1989). The branches of damaged pine trees become wrinkled due to water loss, resin oozes from the trunk, and infection at the base of the trunk causes bark rot. The infection leads to the death of the whole plant. *Cronartium quercuum* infects the trunk, lateral branches, and bare roots of pine trees and forms galls, which seriously affect the seed-setting ability of *P. massoniana* (Li et al. 1995). Zhuang (2001) recorded the pathogen *Diplodia sapinea* (Fr.) P. Karst., which causes shoot blight of *P. massoniana*. *Fusarium oxysporum* Schltdl. can cause damping-off of *P. massoniana* (Luo and Yu 2020). Wilt disease, also known as damping-off disease, begins with the withering of the top needles and, over time, leads to root rot until the whole seedling dies. This pathogen has been proven to cause plant withering (Tint 1945) and root rot (Bloomberg 1971). *Pseudofusicoccum kimberleyense* Pavlic, T.I. Burgess & M.J. Wingf. and *Pse. violaceum* Mehl & Slippers can cause *P. massoniana* branch blight (Li et al. 2023). Needle blight of *P. massoniana* is caused by *Pestalotiopsis funerea* (Desm.) Steyaert, which makes the needles dry up, die, and defoliate early (Liang et al. 2002).

As endophytes, plant pathogens, or saprophytes, *Pestalotiopsis* species are widely distributed throughout the world, mainly in tropical and temperate regions, and have a wide range of host plants (Bate-Smith and Metcalfe 1957; Guba 1961; Sutton 1980; Jeewon et al. 2003; Maharachchikumbura et al. 2011). *Pestalotiopsis* Steyaert was segregated from *Pestalotia* by Steyaert (1949). Its stable characteristics include the length and width of conidia, the length and color of the median three cells, and the number and length of apical appendages (Jeewon et al. 2002). However, excessive overlap of conidia makes it difficult to identify species solely by morphological characteristics (Maharachchikumbura et al. 2011). Although some additional taxonomic features can be used as identification bases for *Pestalotiopsis* species—such as pigmentation of intermediate cells, an important feature that distinguishes *P. funerea* from *Pestalotiopsis triseta* (Moreau & M. Moreau) Steyaert—they still have great limitations (Griffiths and Swart 1974). In the past, Steyaert (1949) and Guba (1961) divided species with variegated conidia into two groups according to the color of intermediate cells. However, with the development of identification technology, phylogenetic analyses based on multi-gene sequences have shown that classifying species by the color of intermediate cells is unreliable (Maharachchikumbura et al. 2014).

As a plant pathogen, needle blight caused by *Pestalotiopsis* is a common disease in young forests and is widespread and seriously detrimental. Li et al. (2024) discovered that *Pestalotiopsis jiangsuensis* is the pathogen of pinprick disease of *P. massoniana*. *Pestalotiopsis funerea* can infect *Pinus tabulaeformis* Carrière (Huang and He 2000), *P. taeda* (Huang 2018), *P. massoniana* (Maharachchikumbura et al. 2014), and other species, causing needle blight. Xu et al. (2017) reported that the pathogen causing needle blight of *P. sylvestris* L. was *Parosela citrina* Rydb. Needle blight not only leads to chlorosis of leaves and withering of branches but, in severe cases, can cause death of the entire plant, resulting in serious economic and ecological losses (Orlikowski et al. 2014; Hu et al. 2020; Monteiro et al. 2022).

In April and July 2023, needle blight samples of *P. massoniana* were collected in Quanjiao County, Anhui Province, and Liuzhou City, Guangxi Province, respectively. Over time, needle blight of *P. massoniana* occurred more frequently, and the pathogen spread quickly, resulting in the invasion of more *P. massoniana* forests. Therefore, the main purpose of this study was to determine the needle blight pathogen of *P. massoniana*, and its pathogenicity was verified by Koch’s postulates.

## ﻿Materials and methods

### ﻿Field survey and fungal isolation

In April and July 2023, pine needle lesions of *P. massoniana* were discovered in Liuzhou City and Quanjiao County, respectively. The entire *P. massoniana* forest was inspected, and samples of diseased needles from five trees were taken to the laboratory for further observation. After macroscopic and microscopic examination of the diseased needles, the healthy and diseased middle parts of the needles were cut with sterile scissors. The surface was disinfected in 70% ethanol for 30 s, in 1% NaClO for 90 s, and then washed three times in sterile water for 90 s each. The pine needle fragments were spread on sterile dry filter paper, dried, and inoculated onto potato dextrose agar (PDA) for 3 days in darkness. The hyphal tips of fungi growing from tissue blocks were cut and transferred to new PDA plates to obtain pure cultures.

### ﻿Morphological identification

Colony morphology and pigment production were observed after the culture was grown at 25 °C for 7 days. During this period, the development of fungal spores was monitored daily. The shape and color of acervuli and conidial masses were observed using a Zeiss stereo microscope (SteRo Discovery V20, Oberkochen, Germany). A Zeiss Axio Imager A2m microscope (Carl Zeiss, Oberkochen, Germany) was used to examine the micromorphology of the strains, including the shape, color, and number of appendages of conidiophores, conidiogenous cells, and conidia.

### ﻿Genomic DNA extraction, PCR, and sequencing

Genomic DNA of the fungi was extracted from the aerial mycelia of 5-day-old cultures using the cetyltrimethylammonium bromide (CTAB) method. Three genomic loci—internal transcribed spacer (ITS), partial translation elongation factor 1-alpha (*TEF1*), and partial β-tubulin (*TUB2*)—were amplified with the primers ITS1/ITS4 (White et al. 1990), EF1-728F/EF1-986R (Carbone and Kohn 1999), and T1/Bt-2b (Glass and Donaldson 1995; O’Donnell and Cigelnik 1997), respectively. The reaction conditions are shown in Table 1. PCR amplifications were performed in a thermal cycler with a 50-μL reaction volume. Each 50-μL PCR reaction contained 25 μL of Premix TaqTM (Takara Biomedical Technology Company Limited, Beijing, China), 2 μL of forward primer, 2 μL of reverse primer, 2 μL of DNA template, and 19 μL of sterile water. The PCR amplicons were purified and sequenced by Sangon Biotech (Shanghai, China).

**Table 1.** Reaction conditions used in PCR amplification and sequencing.

Locus	PCR primers (forward/reverse)	PCR thermal cycles (annealing temperature in bold)
ITS	ITS5/ITS4	94 °C: 3 min, (94 °C: 45 s, 55 °C: 45 s, 72 °C: 1 min) ×35 cycles, 72 °C: 10 min
*TEF1*	EF1-728F/EF1-986R	94 °C: 3 min, (94 °C: 45 s, 55 °C: 45 s, 72 °C: 1 min) ×35 cycles, 72 °C: 10 min
*TUB2*	T1/Bt-2b	94 °C: 3 min, (94 °C: 45 s, 56 °C: 60 s, 72 °C: 1 min) ×35 cycles, 72 °C: 10 min

### ﻿Phylogenetic analyses

Based on partial comparison results from the NCBI GenBank nucleotide database and recent studies on *Pestalotiopsis* species, reference strains were selected. *Neopestalotiopsis protearum* (CBS 114178) was designated as the outgroup taxon. Concatenated multilocus data (ITS, *TEF1*, and *TUB2*) were used for phylogenetic analyses with maximum likelihood (ML) and Bayesian inference (BI). MAFFT version 7.313 (Katoh and Standley 2013) and BioEdit version 7.0.9.0 (Hall 1999) were used to manually align and edit DNA sequences. IQ-TREE version 1.6.8 (Nguyen et al. 2015) was used to conduct ML analyses on the multilocus alignments. The GTR+F+I+G4 substitution model and bootstrap method were applied, with 1,000 replications to infer phylogenetic relationships. RAxML bootstrap support values were considered significant at ML ≥ 70. Bayesian inference was performed using MrBayes version 3.2.6 (Quaedvlieg et al. 2014) under the GTR+I+G+F model (two parallel runs; 2,000,000 generations). Bayesian posterior probability values were considered significant at PP ≥ 0.90. The phylogenetic tree was constructed in FigTree version 1.4.4 (http://tree.bio.ed.ac.uk/software/figtree/).

### ﻿Genealogical concordance phylogenetic species recognition analyses

SplitsTree version 4.14.6 and Genealogical Concordance Phylogenetic Species Recognition (GCPSR) analysis were used to assess recombination levels among closely related species. The pairwise homoplasy index (PHI) test was performed, and the new taxon of *Pestalotiopsis* and its most closely related species were independently evaluated. A PHI value (Φw < 0.05) indicates significant recombination in the dataset. Using the LogDet transform and splits decomposition options, the relationships among closely related species were displayed in a splits diagram.

### ﻿Pathogenicity test

The pathogenicity of 10 representative isolates was tested on 33 healthy 2-year-old *P. massoniana* seedlings. The tested plants were obtained from the Gudong Seedling Base in Hechi, Guangxi, China. Healthy needles of *P. massoniana* were stabbed with sterile needles, each producing one wound, and conidial suspensions (10^6^ conidia·mL^−1^) were sprayed evenly on the wounds. Each isolate was inoculated onto three plants, while control plants were sprayed with sterile water. Inoculated and control seedlings were placed in a chamber (1.5 × 1.2 × 1.5 m) equipped with a humidifier (300 mL/h) to maintain relative humidity (RH) at 70%. The chamber was kept in a greenhouse at 25 ± 2 °C and observed continuously for 10 days. All experiments were conducted three times.

## ﻿Results

### ﻿Disease symptoms and fungal isolation

**Figure 1.**
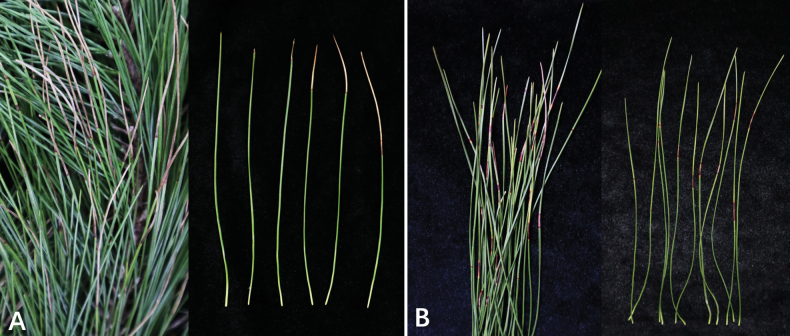
Symptoms of needle blight of *Pinus massoniana* found in Quanjiao County, Anhui Province (A), and Liuzhou City, Guangxi Province (B).

In April and July 2023, needle blight of *P. massoniana* was observed in Liuzhou City and Quanjiao County, showing two distinct symptom types. In Liuzhou, the disease presented as necrotic bands and dead areas, with necrotic bands encircling pine needles of varying sizes. These bands were dry and discolored, not easily broken, and the pine needles outside the necrotic zones remained green (Fig. 1B), with an incidence rate of approximately 70%. However, in Quanjiao County, the disease symptoms included dead needle tips or upper parts of needles that broke off easily, while the remaining portions of the needles stayed green (Fig. 1A), with an incidence rate of approximately 70%. The two sample sets from Liuzhou City and Quanjiao County were treated separately. Based on colony morphology on PDA and ITS sequence analysis, 183 *Pestalotiopsis* strains were isolated and identified from the two regions, with isolation frequencies of 91% and 92%, respectively. Ten representative isolates (DB 1-1, DB 1-2, DB 1-3, DB 1-4, DB 1-5, AH 1-1, AH 1-2, AH 1-3, AH 1-4, and AH 1-5) were selected for further study and deposited at the China Forestry Culture Collection Center (CFCC).

### ﻿Phylogenetic analyses

**Figure 2.**
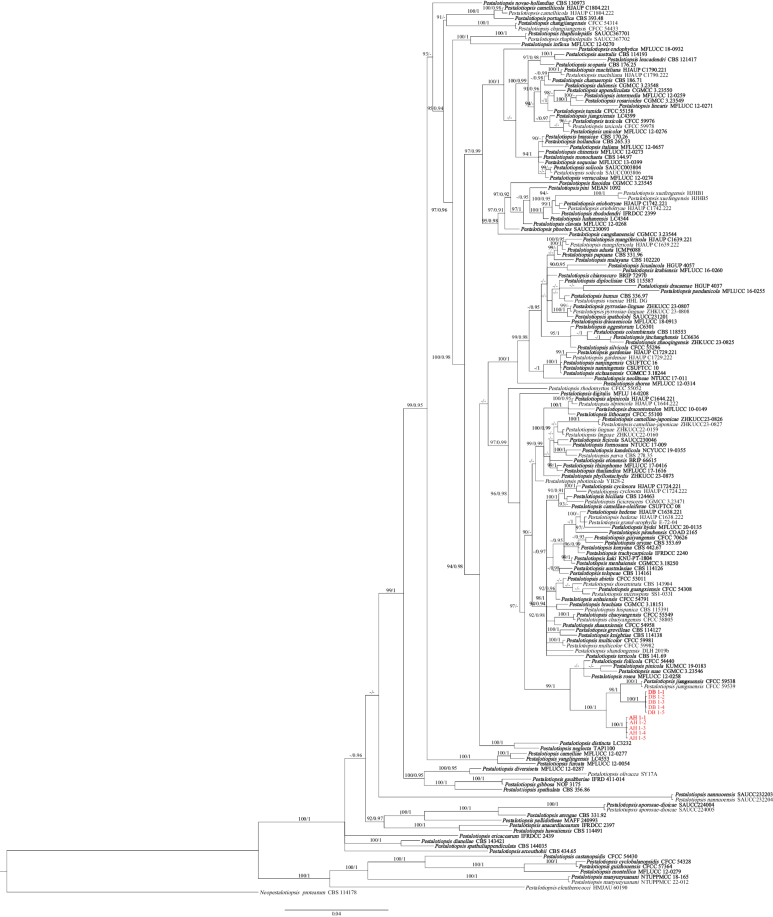
Phylogenetic relationships of *Pestalotiopsis* isolates DB 1-1, DB 1-2, DB 1-3, DB 1-4, DB 1-5, AH 1-1, AH 1-2, AH 1-3, AH 1-4, and AH 1-5, based on concatenated sequences of ITS, *TEF1*, and *TUB2* regions. RAxML bootstrap support values (ML ≥ 70) and Bayesian posterior probability values (PP ≥ 0.90) are shown at the nodes (ML/PP). *Neopestalotiopsis protearum* (CBS 114178) was used as the outgroup. Scale bar = 0.04 substitutions per nucleotide position. Sequences from this study are shown in red, and ex-type strains are shown in bold.

**Figure 3.**
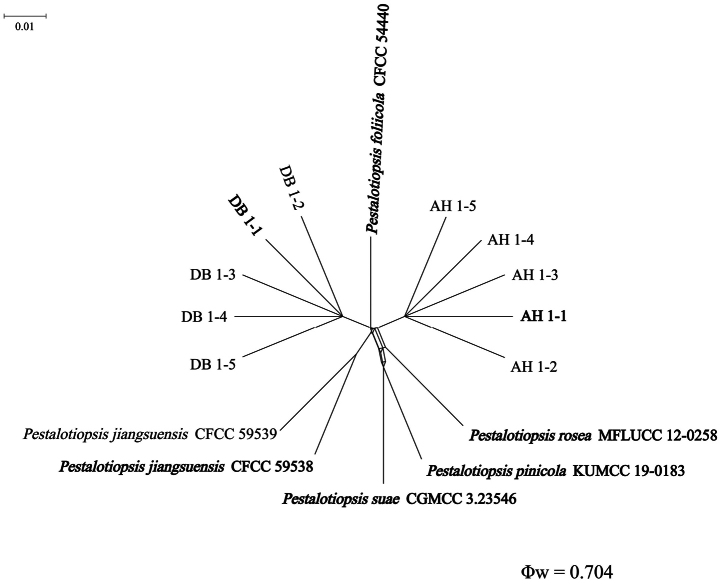
Pairwise homoplasy index (PHI) test of *Pestalotiopsis* isolates DB 1-1, DB 1-2, DB 1-3, DB 1-4, DB 1-5, AH 1-1, AH 1-2, AH 1-3, AH 1-4, and AH 1-5, and closely related *P. foliicola*, *P. pinicola*, *P. suae*, *P. rosea*, and *P. jiangsuensis*, using both LogDet transformation and splits decomposition. PHI test results (Φw < 0.05) indicate significant recombination within the dataset.

The ten representative isolates, together with 163 additional isolates and *Neopestalotiopsis protearum* CBS 114178 as the outgroup, were included in multilocus phylogenetic analyses using concatenated ITS, *TEF1*, and *TUB2* sequences (Table 2; Fig. 2). A total of 1,801 bp, including gaps, was obtained (ITS = 1–657, *TEF1* = 658–1,273, *TUB2* = 1,274–1,801). The tree topologies generated from the ML and BI analyses were consistent, with ML bootstrap support values greater than 70% and Bayesian posterior probabilities (BPP) greater than 0.90 shown at the nodes (ML/BI). In the phylogenetic analyses, the ten isolates were clustered into two independent clades (ML/BI = 100/1), which formed a larger branch with four ex-type strains with strong support (ML/BI = 100/1: *Pestalotiopsis foliicola*CFCC 54440, *P. pinicola* KUMCC 19-0183, *P. suae* CGMCC3.23546, and *P. rosea* MFLUCC 12-0258). DB 1-1, DB 1-2, DB 1-3, DB 1-4, DB 1-5, and *P. jiangsuensis*CFCC 59538 (ex-type) clustered into a smaller branch with strong support (ML/BI = 100/1), while AH 1-1, AH 1-2, AH 1-3, AH 1-4, and AH 1-5 were placed in a different clade (Fig. 2). Based on the three-locus phylogenetic analyses and morphology, ten strains (DB 1-1, DB 1-2, DB 1-3, DB 1-4, DB 1-5, AH 1-1, AH 1-2, AH 1-3, AH 1-4, and AH 1-5) were identified as two different new species of *Pestalotiopsis*. The PHI test of the new species showed no significant recombination (Φw = 0.704) with their closely related taxa (*P. foliicola*, *P. pinicola*, *P. suae*, *P. rosea*, and *P. jiangsuensis*) (Fig. 3)

**Table 2.** Host, origin, and GenBank accession numbers of strains of *Pestalotiopsis* species used for phylogenetic analyses.

Species	Strain number^b^	Host	Origin	GenBank accession number^c^		
				ITS	TUB2	TEF
*Pestalotiopsis abietis*	CFCC 53011^T^	*Abies fargesii*	China	MK397013	MK622280	MK622277
*P. adusta*	ICMP 6088^T^	*Prunus cerasus*	Fiji	JX399006	JX399037	JX399070
*P. aggestorum*	LC6301^T^	*Camellia sinensis*	China	KX895015	KX895348	KX895234
*P. alpinicola*	HJAUP C1644.221^T^	*Alpinia zerumbet*	China	PP962274	PP952219	PP952249
*P. alpinicola*	HJAUP C1644.222	*Alpinia zerumbet*	China	PP962275	PP952220	PP952248
*P. anacardiacearum*	IFRDCC 2397^T^	*Mangifera indica*	China	KC247154	KC247155	KC247156
*P. anhuiensis*	CFCC 54791^T^	*Cyclobalanopsis glauca*	China	ON007028	ON005056	ON005045
*P. aporosae-dioicae*	SAUCC224004^T^	*Aporosa dioica*	China	OR733506	OR912985	OR912988
*P. aporosae-dioicae*	SAUCC224005	*Aporosa dioica*	China	OR733505	OR912986	OR912989
*P. appendiculate*	CGMCC 3.23550^T^	*Rhododendron decorum*	China	OP082431	OP185516	OP185509
*P. arengae*	CBS 331.92^T^	*Arenga undulatifolia*	Singapore	KM199340	KM199426	KM199515
*P. arceuthobii*	CBS 434.65^T^	*Arceuthobium campylopodum*	USA	KM199341	KM199427	KM199516
*P. australasiae*	CBS 114126^T^	*Knightia* sp.	New Zealand	KM199297	KM199409	KM199499
*P. australis*	CBS 114193^T^	*Grevillea* sp.	Australia	KM199332	KM199383	KM199475
*P. biciliate*	CBS 124463^T^	*Platanus × hispanica*	Slovakia	KM199308	KM199399	KM199505
*P. brachiate*	CGMCC 3.18151^T^	*Rhizophora apiculata*	Thailand	MK764274	MK764340	MK764318
*P. brassicae*	CBS 170.26^T^	*Brassica napus*	New Zealand	KM199379	-	KM199558
*P. camelliae*	MFLUCC 12-0277^T^	*Camellia japonica*	China	JX399010	JX399041	JX399074
*P. camelliae-japonicae*	ZHKUCC23-0826^T^	*Camellia japonica*	China	OR258040	OR251483	OR251480
*P. camelliae-japonicae*	ZHKUCC23-0827	*Camellia japonica*	China	OR258041	OR251484	OR251481
*P. camelliae-oleiferae*	CSUFTCC 08^T^	*Camellia oleifera*	China	OK493593	OK562368	OK507963
*P. camelliicola*	HJAUP C1804.221^T^	*Camellia japonica*	China	PP962357	PP952229	PP952236
*P. camelliicola*	HJAUP C1804.222	*Camellia japonica*	China	PP962358	PP952230	PP952235
*P. cangshanensis*	CGMCC 3.23544^T^	*Rhododendron delavayi*	China	OP082426	OP185517	OP185510
*P. castanopsidis*	CFCC 54430^T^	*Castanopsis lamontii*	China	OK339732	OK358508	OK358493
*P. chamaeropis*	CBS 186.71^T^	*Chamaerops humilis*	Italy	KM199326	KM199391	KM199473
*P. changjiangensis*	CFCC 54314^T^	*Castanopsis tonkinensis*	China	OK339739	OK358515	OK358500
*P. changjiangensis*	CFCC 54433	*Castanopsis tonkinensis*	China	OK339740	OK358516	OK358501
*P. chaoyangensis*	CFCC 55549^T^	*Euonymus japonicus*	China	OQ344763	OQ410584	OQ410582
*P. chaoyangensis*	CFCC 58805	*Euonymus japonicus*	China	OQ344764	OQ410585	OQ410583
*P. chiaroscuro*	BRIP 72970^T^	*Sporobolus natalensis*	Australia	OK422510	-	-
*P. chinensis*	MFLUCC 12-0273^T^	*Taxus* sp.	China	JX398995	-	-
*P. clavate*	MFLUCC 12-0268^T^	*Buxus* sp.	China	JX398990	JX399025	JX399056
*P. colombiensis*	CBS 118553^T^	*Eucalyptus urograndis*	Colombia	KM199307	KM199421	KM199488
*P. cyclobalanopsidis*	CFCC 54328^T^	*Cyclobalanopsis glauca*	China	OK339735	OK358511	OK358496
*P. cyclosora*	HJAUP C1724.221^T^	*Cyclosorus interruptus*	China	PP962279	PP952221	PP952247
*P. cyclosora*	HJAUP C1724.222	*Cyclosorus interruptus*	China	PP962280	PP952222	PP952246
*P. daliensis*	CGMCC 3.23548^T^	*Rhododendron decorum*	China	OP082429	OP185511	OP185518
*P. dianellae*	CBS 143421^T^	*Dianella* sp.	Australia	MG386051	MG386164	-
*P. digitalis*	MFLU 14-0208^T^	*Digitalis purpurea*	New Zealand	KP781879	KP781883	-
*P. diploclisiae*	CBS 115587^T^	*Diploclisia glaucescens*	China	KM199320	KM199419	KM199486
*P. disseminate*	CBS 143904	*Persea americana*	New Zealand	MH554152	MH554825	MH554587
*P. distincta*	LC3232^T^	*Camellia sinensis*	China	KX894961	KX895293	KX895178
*P. diversiseta*	MFLUCC12-0287^T^	*Rhododendron* sp.	China	JX399009	JX399040	JX399073
*P. dracaenae*	HGUP 4037^T^	*Dracaena fragrans*	China	MT596515	MT598645	MT598644
*P. dracaenicola*	MFLUCC 18-0913^T^	*Dracaena* sp.	Thailand	MN962731	MN962733	MN962732
*P. dracontomelon*	MFLUCC 10-0149^T^	*Dracontomelon dao*	Thailand	KP781877	-	KP781880
*P. eleutherococci*	HMJAU 60190	*Eleutherococcus brachypus*	China	OL996127	OL898722	-
*P. endophytica*	MFLUCC 18-0932^T^	*Magnolia garrettii*	Thailand	MW263946	-	MW417119
*P. ericacearum*	IFRDCC 2439^T^	*Rhododendron delavayi*	China	KC537807	KC537821	KC537814
*P. eriobotryae*	HJAUP C1742.221^T^	*Eriobotrya japonica*	China	PP962289	PP952227	PP952238
*P. eriobotryae*	HJAUP C1742.222	*Eriobotrya japonica*	China	PP962291	PP952228	PP952237
*P. etonensis*	BRIP 66615^T^	*Sporobolus jacquemontii*	Australia	MK966339	MK977634	MK977635
*P. ficicola*	SAUCC230046^T^	*Ficus microcarpa*	China	OQ691974	OQ718749	OQ718691
*P. ficicrescens*	CGMCC 3.23471	Oleaceae	China	OR381055	OR247980	OR361455
*P. foliicola*	CFCC 54440^T^	*Castanopsis faberi*	China	ON007029	ON005057	ON005046
*P. formosana*	NTUCC 17-009^T^	*Neolitsea villosa*	China	MH809381	MH809385	MH809389
*P. furcate*	MFLUCC 12-0054^T^	*Camellia sinensis*	Thailand	JQ683724	JQ683708	JQ683740
*P. fusoidea*	CGMCC 3.23545^T^	*Rhododendron delavayi*	China	OP082427	OP185519	OP185512
*P. gardenia*	HJAUP C1729.221^T^	*Gardenia jasminoides*	China	PP962285	PP952225	PP952241
*P. gardenia*	HJAUP C1729.222	*Gardenia jasminoides*	China	PP962286	PP952226	PP952240
*P. gaultheriae*	IFRD 411-014^T^	*Gaultheria forrestii*	China	KC537805	KC537819	KC537812
*P. gibbosa*	NOF 3175^T^	*Gaultheria shallon*	Canada	LC311589	LC311590	LC311591
*P. grandis-urophylla*	E72-04	*Eucalyptus grandis*	Brazil	KU926710	KU926718	KU926714
*P. grevilleae*	CBS 114127^T^	*Grevillea* sp.	Australia	KM199300	KM199407	KM199504
*P. guangxiensis*	CFCC 54308^T^	*Quercus griffithii*	China	OK339737	OK358513	OK358498
*P. guiyangensis*	CFCC 70626^T^	*Eriobotrya japonica*	China	PP784740	PP842617	PP842629
*P. guizhouensis*	CFCC 57364^T^	*Cyclobalanopsis glauca*	China	ON007035	ON005063	ON005052
*P. hawaiiensis*	CBS 114491^T^	*Leucospermum* sp.	USA	KM199339	KM199428	KM199514
*P. hederae*	HJAUP C1638.221^T^	*Hedera helix*	China	PP962270	PP952252	PP952234
*P. hederae*	HJAUP C1638.222	*Hedera helix*	China	PP962271	PP952216	-
*P. hispanica*	CBS 115391	*Eucalyptus globulus*	Portugal	MW794107	MW802840	MW805399
*P. hollandica*	CBS 265.33^T^	*Sciadopitys verticillata*	Netherlands	KM199328	KM199388	KM199481
*P. humus*	CBS 336.97^T^	Soil	Papua New Guinea	KM199317	KM199420	KM199484
*P. hydei*	MFLUCC 20-0135^T^	*Litsea petiolata*	Thailand	MW266063	MW251112	MW251113
*P. inflexa*	MFLUCC 12-0270^T^	Unidentified tree	China	JX399008	JX399039	JX399072
*P. intermedia*	MFLUCC 12-0259^T^	Unidentified tree	China	JX398993	JX399028	JX399059
*P. italiana*	MFLUCC 12-0657^T^	*Cupressus glabra*	Italy	KP781878	KP781882	KP781881
*P. jiangsuensis*	CFCC 59538^T^	*Pinus massoniana*	China	OR533577	OR539191	OR539186
*P. jiangsuensis*	CFCC 59539	*Pinus massoniana*	China	OR533578	OR539192	OR539187
*P. jiangxiensis*	LC4399^T^	*Camellia* sp.	China	KX895009	KX895341	KX895227
*P. jinchanghensis*	LC6636^T^	*Camellia sinensis*	China	KX895028	KX895361	KX895247
*P. kaki*	KNU-PT-1804^T^	*Diospyros kaki*	Korea	LC552953	LC552954	LC553555
*P. kandelicola*	NCYUCC 19-0355^T^	*Kandelia candel*	China	MT560723	MT563100	MT563102
***P. kendrickii***	**AH 1-1^T^**	***Pinus massoniana***	**China**	**PP764798**	**PP764175**	**PP764170**
	**AH 1-2**			**PP764799**	**PP764176**	**PP764171**
	**AH 1-3**			**PP764800**	**PP764177**	**PP764172**
	**AH 1-4**			**PP764801**	**PP764178**	**PP764173**
	**AH 1-5**			**PP764802**	**PP764179**	**PP764174**
*P. kenyana*	CBS 442.67^T^	*Coffea* sp.	Kenya	KM199302	KM199395	KM199502
*P. knightiae*	CBS 114138^T^	*Knightia* sp.	New Zealand	KM199310	KM199408	KM199497
*P. krabiensis*	MFLUCC 16-0260^T^	*Pandanus* sp.	Thailand	MH388360	MH412722	MH388395
*P. leucadendri*	CBS 121417^T^	*Leucadendron* sp.	South Africa	MH553987	MH554654	MH554412
*P. licualacola*	HGUP4057^T^	*Licuala grandis*	China	KC492509	KC481683	KC481684
*P. linearis*	MFLUCC 12-0271^T^	*Trachelospermum* sp.	China	JX398992	JX399027	JX399058
*P. linguae*	ZHKUCC 22-0159	*Pyrrosia lingua*	China	OP094104	OP186108	OP186110
*P. linguae*	ZHKUCC 22-0160	*Pyrrosia lingua*	China	OP094103	OP186107	OP186109
*P. lithocarpi*	CFCC 55100^T^	*Lithocarpus chiungchungensis*	China	OK339742	OK358518	OK358503
***P. liuzhouensis***	**DB 1-1^T^**	***Pinus massoniana***	**China**	**PP766224**	**PP764185**	**PP764180**
	**DB 1-2**			**PP766225**	**PP764186**	**PP764181**
	**DB 1-3**			**PP766226**	**PP764187**	**PP764182**
***P. liuzhouensis***	**DB 1-4**	***Pinus massoniana***	**China**	**PP766227**	**PP764188**	**PP764183**
	**DB 1-5**			**PP766228**	**PP764189**	**PP764184**
*P. lushanensis*	LC4344^T^	*Camelia* sp.	China	KX895005	KX895337	KX895223
*P. machiliana*	HJAUP C1790.221^T^	*Machilus pauhoi*	China	PP962355	PP952214	PP952253
*P. machiliana*	HJAUP C1790.222	*Machilus pauhoi*	China	PP962356	PP952215	PP952254
*P. malayana*	CBS 102220^T^	*Macaranga triloba*	Malaysia	KM199306	KM199411	KM199482
*P. mangifericola*	HJAUP C1639.221^T^	*Mangifera indica*	China	PP962272	PP952217	PP952251
*P. mangifericola*	HJAUP C1639.222	*Mangifera indica*	China	PP962273	PP952218	PP952250
*P. manyueyuanani*	NTUPPMCC 18-165^T^	*Ophiocordyceps* sp.	China	OR125060	OR126306	OR126313
*P. manyueyuanani*	NTUPPMCC 22-012	*Ophiocordyceps* sp.	China	OR125061	OR126307	OR126314
*P. menhaiensis*	CGMCC 3.18250^T^	*Camellia sinensis*	China	KU252272	KU252488	KU252401
*P. microspora*	SS1-033I	*Cornus canadensis*	Canada	MT644300	-	-
*P. monochaeta*	CBS 144.97^T^	*Quercus robur*	Netherlands	KM199327	KM199386	KM199479
*P. montellica*	MFLUCC12-0279^T^	*Fagraea bodeni*	China	JX399012	JX399043	JX399076
*P. multicolor*	CFCC 59981^T^	Chinese yew	China	OQ626676	OQ714336	OQ714341
*P. multicolor*	CFCC 59982	Chinese yew	China	OQ771896	OQ779488	OQ779483
*P. nanjingensis*	CSUFTCC 16^T^	*Camellia oleifera*	China	OK493602	OK562377	OK507972
*P. nanningensis*	CSUFTCC 10^T^	*Camellia oleifera*	China	OK493596	OK562371	OK507966
*P. nannuoensis*	SAUCC232203^T^	-	China	OR733504	OR912991	OR863909
*P. nannuoensis*	SAUCC232204	-	China	OR733503	OR912992	OR863910
*P. neglecta*	TAP1100^T^	*Quercus myrsinaefolia*	Japan	AB482220	LC311599	LC311600
*P. neolitseae*	NTUCC 17-011^T^	*Neolitsea villosa*	China	MH809383	MH809387	MH809391
*P. novae-hollandiae*	CBS 130973^T^	*Banksia grandis*	Australia	KM199337	KM199425	KM199511
*P. olivacea*	SY17A	*Pinus armandii*	China	EF055215	EF055251	-
*P. oryzae*	CBS 353.69^T^	*Oryza sativa*	Denmark	KM199299	KM199398	KM199496
*P. pallidotheae*	MAFF 240993^T^	*Pieris japonica*	Japan	AB482220	LC311584	LC311585
*P. pandanicola*	MFLUCC 16-0255^T^	*Pandanus* sp.	Thailand	MH388361	MH412723	MH388396
*P. papuana*	CBS 331.96^T^	Coastal soil	Papua New Guinea	KM199321	KM199413	KM199491
*P. parva*	CBS 278.35	*Leucothoe fontanesiana*	Thailand	KM199313	KM199405	KM199509
*P. phoebes*	SAUCC230093^T^	*Phoebe zhenna*	China	OQ692028	OQ718803	OQ718745
*P. photinicola*	YB28-2	Mango	China	MK228997	MK360938	MK512491
*P. phyllostachydis*	ZHKUCC 23-0873^T^	-	China	OR343210	OR367676	OR367675
*P. pini*	MEAN 1092^T^	*Pinus pinea*	Portugal	MT374680	MT374705	MT374693
*P. pinicola*	KUMCC 19-0183^T^	*Pinus armandii*	China	MN412636	MN417507	MN417509
*P. piraubensis*	COAD 2165^T^	*Psidium guajava*	Brazil	MH627381	MH643773	MH643774
*P. portugallica*	CBS 393.48^T^	-	Portugal	KM199335	KM199422	KM199510
*P. pyrrosiae-linguae*	ZHKUCC 23-0807^T^	*Pyrrosia lingua*	China	OR199902	OR259258	OR259260
*P. pyrrosiae-linguae*	ZHKUCC 23-0808	*Pyrrosia lingua*	China	OR199903	OR259259	OR259261
*P. rhaphiolepidis*	SAUCC367701^T^	*Rhaphiolepis indica*	China	OR733502	OR863906	OR912994
*P. rhaphiolepidis*	SAUCC367702	*Rhaphiolepis indica*	China	OR733501	OR863907	OR912995
*P. rhizophorae*	MFLUCC 17-0416^T^	*Rhizophora apiculata*	Thailand	MK764283	MK764349	MK764327
*P. rhododendri*	IFRDCC 2399^T^	*Rhododendron sinogrande*	China	KC537804	KC537818	KC537811
*P. rhodomyrtus*	CFCC 55052	*Cyclobalanopsis augustinii*	China	OM746311	OM839984	OM840083
*P. rosarioides*	CGMCC 3.23549^T^	*Rhododendron decorum*	China	OP082430	OP185513	OP185520
*P. rosea*	MFLUCC 12-0258^T^	*Pinus* sp.	China	JX399005	JX399036	JX399069
*P. scoparia*	CBS 176.25^T^	*Chamaecyparis* sp.	China	KM199330	KM199393	KM199478
*P. sequoia*	MFLUCC 13-0399^T^	*Sequoia sempervirens*	Italy	KX572339	-	-
*P. shaanxiensis*	CFCC 54958^T^	*Quercus variabilis*	China	ON007026	ON005054	ON005043
*P. shandongensis*	DLH 2019a	*Rosa chinensis*	China	MN625276	MN626730	MN626741
*P. shorea*	MFLUCC 12-0314^T^	*Shorea obtusa*	Thailand	KJ503811	KJ503814	KJ503817
*P. sichuanensis*	CGMCC 3.18244^T^	*Camellia sinensis*	China	KX146689	KX146807	KX146748
*P. silvicola*	CFCC 55296^T^	*Cyclobalanopsis kerrii*	China	ON007032	ON005060	ON005049
*P. solicola*	SAUCC003804^T^	Soil	China	OQ692020	OQ718795	OQ718737
*P. solicola*	SAUCC003806	Soil	China	OQ692021	OQ718796	OQ718738
*P. spatholobi*	SAUCC231201^T^	*Spatholobus suberectus*	China	OQ692023	OQ718798	OQ718740
*P. spathulate*	CBS 356.86^T^	*Gevuina avellana*	Chile	KM199338	KM199423	KM199513
*P. spathuli appendiculata*	CBS 144035^T^	*Phoenix canariensis*	Australia	MH554172	MH554845	MH554607
*P. suae*	CGMCC3.23546^T^	*Rhododendron delavayi*	China	OP082428	OP185521	OP185514
*P. taxicola*	CFCC 59976^T^	Chinese yew	China	OQ626673	OQ714333	OQ714338
*P. taxicola*	CFCC 59978	Chinese yew	China	OQ771893	OQ779485	OQ779480
*P. telopeae*	CBS 114161^T^	*Telopea* sp.	Australia	KM199296	KM199403	KM199500
*P. terricola*	CBS 141.69^T^	Soil	Pacific Islands	MH554004	MH554680	MH554438
*P. thailandica*	MFLUCC 17-1616^T^	*Rhizophora apiculata*	Thailand	MK764285	MK764351	MK764329
*P. trachycarpicola*	IFRDCC 2240^T^	*Trachycarpus fortunei*	China	JQ845947	JQ845945	JQ845946
*P. tumida*	CFCC 55158^T^	*Rosa chinensis*	China	OK560610	OL814524	OM158174
*P. unicolor*	MFLUCC 12-0276^T^	*Rhododendron* sp.	China	JX398999	JX399030	-
*P. verruculosa*	MFLUCC 12-0274^T^	*Rhododendron* sp.	China	JX398996	-	JX399061
*P. vismiae*	HHL-DG	*Rhizophora stylosa*	China	HM535704	HM573246	-
*P. xuefengensis*	HJHB1	*Polygonatum cyrtonema*	China	OQ711603	OQ737677	OQ737676
*P. xuefengensis*	HJHB5	*Polygonatum cyrtonema*	China	OQ746334	OQ772277	OQ772275
*P. yanglingensis*	LC4553^T^	*Camellia sinensis*	China	KX895012	KX895345	KX895231
*P. zhaoqingensis*	ZHKUCC 23-0825^T^	-	China	OR233336	OR239062	OR239061
*Neopestalotiopsis protearum*	CBS 114178^T^	*Leucospermum cuneiforme*	Zimbabwe	JN712498	KM199463	LT853201

^a^ Strains isolated from the current study are in bold. ^T^ = ex-type culture. ^b^CFCC = China Forestry Culture Collection Center, China; ICMP = International Collection of Microorganisms from Plants, Auckland, New Zealand; LC = Working collection of Lei Cai, housed at the Institute of Microbiology, Chinese Academy of Sciences, Beijing, China; IFRDCC = International Fungal Research and Development Culture Collection, Kunming, Yunnan China; CGMCC = China General Microbiological Culture Collection Center, Beijing, China; CBS = Culture collection of the Westerdijk Fungal Biodiversity Institute, Utrecht, The Netherlands; MFLUCC = Mae Fah Luang University Culture Collection, Chiang Rai, Thailand; CSUFTCC = Central South University of Forestry and Technology Culture Collection, Hunan, China; BRIP = Plant Pathology Herbarium, Department of Employment, Economic, Development and Innovation, Queensland, Australia; MFLU = Mae Fah Luang University Herbarium, Thailand; HGUP = Plant Pathology Herbarium of Guizhou University, Guizhou, China; HJAUP = Herbarium of Jiangxi Agricultural University, Plant Pathology, Nanchang, China; HMJAU = Herbarium of Mycology of Jilin Agricultural University, Jilin, China; SAUCC = Shandong Agricultural University Culture Collection, Taian, Shandong, China; NTUCC = The Department of Plant Pathology and Microbiology, National Taiwan University Culture Collection, Taipei, Taiwan (ROC); NOF = The Fungus Culture Collection of the Northern Forestry Centre, Alberta, Canada; E = The “Coleção de culturas de fungos fitopatogênicos Prof. Maria Menezes”, Universidade Federal Rural de Pernambuco, Recife, Brazil; KNU = Kyungpook National University, Daegu, South Korea; NCYUCC = The National Chiayi University Culture Collection, Jiayi, Taiwan; ZHKUCC = The culture collection of Zhongkai University of Agriculture and Engineering, Guangzhou City, Guangdong, China; TAP = Tamagawa University, Tokyo, Japan; MAFF = Ministry of Agriculture, Forestry and Fisheries, Tsukuba, Ibaraki, Japan; MEAN = Instituto Nacional de Investigação Agrária e Veterinária I. P.; KUMCC = Kunming Institute of Botany Culture Collection, Yunnan, China. c ITS = internal transcribed spacer; *TUB2* = b-tubulin 2; *TEF1* = translation elongation factor 1-α.

### ﻿Taxonomy

#### ﻿ Pestalotiopsis liuzhouensis

Taxon classificationFungiAmphisphaerialesPestalotiopsidaceae

Li-Hua Zhu, Hui Li & D.W. Li sp. nov.

EBF4DCFB-A9B6-5BC6-9A60-861D37D86DCF

Fungal Names No: FN 572916

Fig. 4

##### Etymology.

the epithet referring to the place where the holotype was collected.

##### Culture characteristics.

On PDA medium, the front of the colony is white, with dense aerial hyphae and complete edges. The center of the back is light yellow.

##### Description.

**Figure 4.**
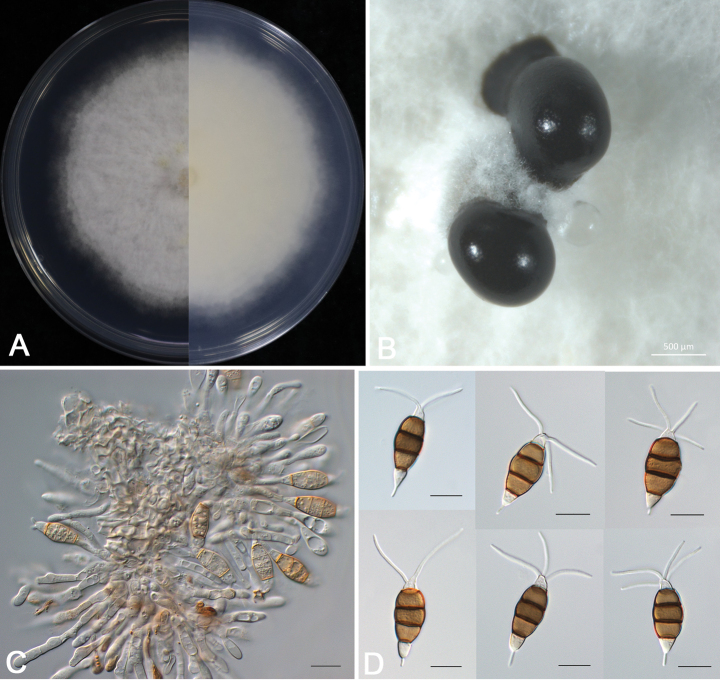
*Pestalotiopsis liuzhouensis* (DB 1-1). A. Colony on PDA after 5 days at 25 °C in the dark; B. Conidiomata and conidial masses; C. Conidiophores, conidiogenous cells, and conidia; D. Conidia. Scale bars: 500 μm (B); 20 μm (C, D).

Sporadic black and gregarious conidiomata produced on PDA after 7 days under light at 25 °C, globose, semi-immersed, dark brown to black, up to 400 μm diam (Fig. 4B); Conidiophores indistinct and reduced to conidiogenous cells. Conidiogenous cells (7.2–)8.5–12(−13.3) × (2.5–)3.1–5.2(−5.9) µm (11.2 ± 1.3 × 3.9 ± 0.7 µm, *n* = 30), hyaline, ampulliform or cylindrical, and sometimes slightly wide at the base (Fig. 4C). Conidia phragmospores, (19.7–)21.1–23.4(–24.8) × (7.8–) 8.4–9.2 (–9.8) µm (22.4 ± 1.4 × 8.9 ± 0.6 µm, *n* = 30), fusoid, ellipsoid, straight to slightly curved, 4-septate (Fig. 4D); basal cell hyaline, obconic, thin-walled, 3.7–6.1 μm long; three median cells doliiform, wall rugose, concolorous, brown, septa darker than the rest of the cell (second cell from the base 3.7–5.6 μm long; third cell 4.1–5.8 μm long; fourth cell 4.0–6.2 μm long); apical cell hyaline, smooth-walled, conic or trapezoid, tapering toward the apex, 2.2–4.4 μm long, with 2–4 tubular apical appendages (mostly 2 and rarely 4), arising from the apical crest, unbranched, filiform, 8.7–23.4 μm long; basal appendage single, tubular, unbranched, centric, 1.5–5.1 μm long.

##### Holotype.

**China** • Guangxi province, Liuzhou city, Shatang Town, Junwu Park, 24°21'45"N, 109°24'07"E (DMS), isolated from needles of *Pinus massoniana*, 23 July 2023, Hui Li, holotype CFCC 70485. Holotype is a living specimen being maintained via lyophilization at the China Forestry Culture Collection Center (CFCC), Chinese Academy of Forestry, Beijing, China, and ex-type DB 1-1 is stored at the Forest Pathology Laboratory, Nanjing Forestry University.

##### Habitat and host.

On needles of *Pinus massoniana* with needle blight.

##### Known distribution.

Liuzhou, Guangxi Province, **China**.

##### Additional specimens examined.

**China** Guangxi province, Liuzhou city, Shatang Town, Junwu Park, 24°21'45"N, 109°24'07"E (DMS), isolated from needles of *Pinus massoniana*, 23 July 2023, Hui Li, cultures: CFCC 70482 (= DB 1-2), CFCC 70479 (= DB 1-3), CFCC 70474 (= DB 1-4), and CFCC 70478 (= DB 1-5).

##### Notes.

Compared with *P. jiangsuensis*, DB 1-1 has wider conidia, 22.4 ± 1.4 × 8.9 ± 0.6 µm vs 23.4 ± 1.8 × 7.5 ± 0.5 μm. The number of apical appendages is 2–4, while there are 1–4 tubular apical appendages in *P. jiangsuensis*.

#### ﻿ Pestalotiopsis kendrickii

Taxon classificationFungiAmphisphaerialesPestalotiopsidaceae

Li-Hua Zhu, Hui Li & D.W. Li sp. nov.

1CD55EBD-75E0-5085-87F1-D513E8C29B50

Fungal Names No: FN 572917

Fig. 5

##### Etymology.

The epithet is named after Dr. Bryce Kendrick to commemorate his contribution to mycology.

##### Culture characteristics.

On PDA medium, the front of the colony is white, with dense aerial hyphae, and the colony slightly bulges in an irregular circle within 1.5 cm from the center, with wavy edges. The center of the back is light yellow, and the color gradually becomes lighter around.

##### Description.

**Figure 5.**
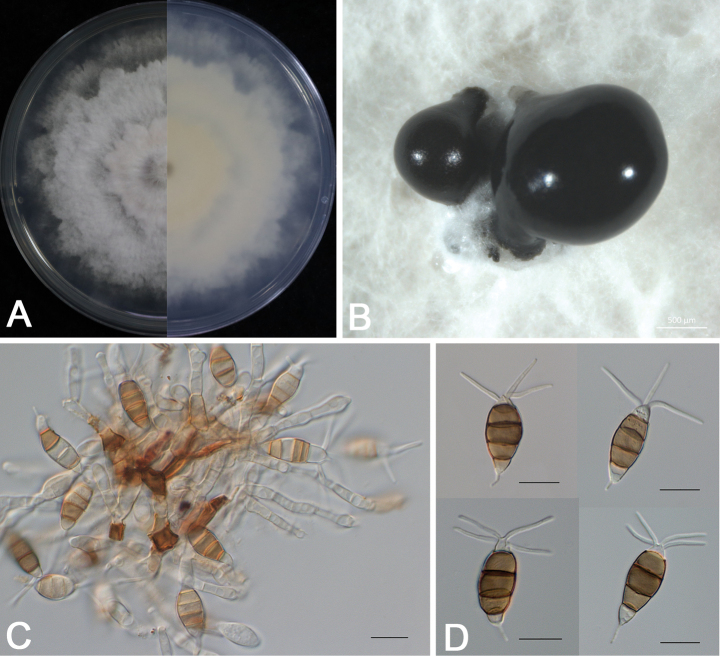
*Pestalotiopsis kendrickii* (AH 1-1). A. Colony on PDA after 5 days at 25 °C in darkness; B. Conidiomata and conidial masses; C. Conidiophores, conidiogenous cells, and conidia; D. Conidia. Scale bars: 500 μm (B); 20 μm (C, D).

Sporadic black and gregarious conidiomata produced on PDA after 8 days under light at 25 °C, globose, semi-immersed, black, up to 1.5 mm diam (Fig. 5B); Conidiophores indistinct and reduced to conidiogenous cells. Conidiogenous cells (5.3–)7.6–11.3(−13.6) × (2.3–)2.8–5.7(−6.2) µm (10.3 ± 2.3 × 4.6 ± 0.5 µm, *n* = 30), hyaline, ampulliform or pear shape, smooth-walled, and sometimes slightly wider at the base (Fig. 5C). Conidia phragmospores, (18.7–)19.8–22.3(–23.4) × (6.5–)7.0–7.8(–8.3) µm (21.1 ± 1.1 × 7.3 ± 0.5 µm, *n* = 30), fusoid, ellipsoid, straight to slightly curved, 4-septate (Fig. 5D); basal cell obconic, hyaline, thin-walled, 3.7–5.5 μm long; three median cells doliiform, wall rugose, light brown, concolorous, septa darker than the rest of the cell (second cell from the base 3.8–5.3 μm long; third cell 3.3–4.6 μm long; fourth cell 3.8–5.3 μm long); apical cell hyaline, smooth-walled, conic or trapezoid, tapering toward the apex, 2.7–3.8 μm long, with 3–4 tubular apical appendages (mostly 3), arising from the apical crest, unbranched, filiform, inserted at the top or side of the apical cell, 10.1–18.7 μm long; basal appendage single, tubular, unbranched, centric, 2.9–6.1 μm long.

##### Holotype.

**China** • Anhui province, Quanjiao city, Washan forest farm, 32°05'06"N, 118°16'22"E (DMS), isolated from needles of *Pinus massoniana*, 26 April 2023, Hui Li, holotype CFCC 70475. Holotype is a living specimen being maintained via lyophilization at the China Forestry Culture Collection Center (CFCC), Chinese Academy of Forestry, Beijing, **China**, and ex-type AH 1-1 is stored at the Forest Pathology Laboratory, Nanjing Forestry University.

##### Habitat and host.

On needles of *Pinus massoniana* with needle blight.

##### Known distribution.

Quanjiao, Anhui Province, **China**.

##### Additional specimens examined.

**China** Anhui province, Quanjiao city, Washan forest farm, 32°05'06"N, 118°16'22"E (DMS), isolated from needles of *Pinus massoniana*, 26 April 2023, Hui Li, cultures: CFCC 70487 (= AH 1-2), CFCC 70495 (= AH 1-3), CFCC 70494 (= AH 1-4), and CFCC 70483 (= AH 1-5).

##### Notes.

*Pestalotiopsis foliicola*, *P. pinicola*, and *P. suae* have 2–3 apical appendages, whereas *P. rosea* has 1–3 tubular apical appendages, some of which are branched. The number of apical appendages in *P. kendrickii* is 3 or 4, and the appendages are unbranched, while *P. jiangsuensis* has 1–4 apical appendages.

##### Pathogenicity test.

**Figure 6.**
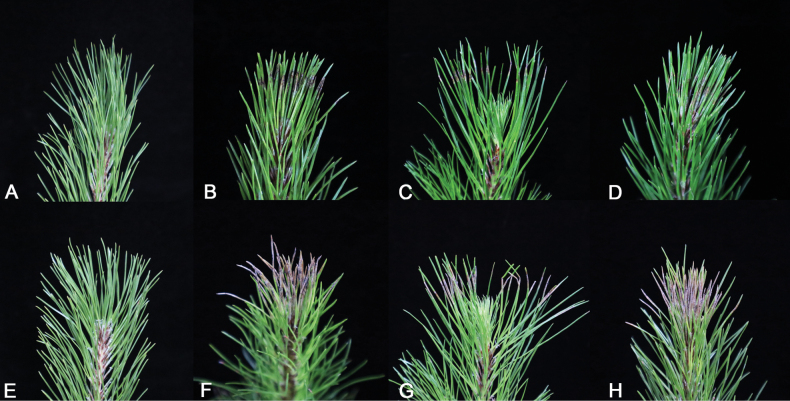
Pathogenicity of representative isolates of *Pestalotiopsis kendrickii* (AH 1-1, AH 1-2, and AH 1-3) on *Pinus massoniana*. A. No symptoms were observed on control pine needles treated with sterile water after 5 days; B–D. Symptoms on pine needles inoculated with conidial suspensions of AH 1-1, AH 1-2, and AH 1-3 after 5 days, respectively; E. No symptoms were observed on control pine needles treated with sterile water after 10 days; F–H. Symptoms on pine needles inoculated with conidial suspensions of AH 1-1, AH 1-2, and AH 1-3 after 10 days.

**Figure 7.**
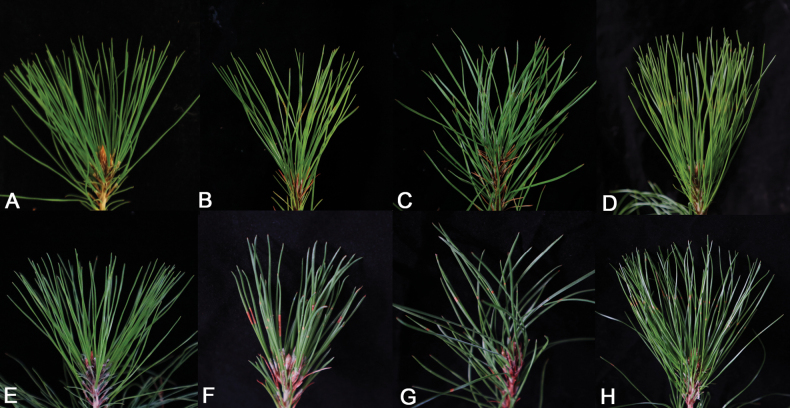
Pathogenicity of representative isolates of *Pestalotiopsis liuzhouensis* (DB 1-1, DB 1-2, and DB 1-3) on *Pinus massoniana*. A. No symptoms were observed on control pine needles treated with sterile water after 5 days; B–D. Symptoms on pine needles inoculated with conidial suspensions of DB 1-1, DB 1-2, and DB 1-3 after 5 days, respectively; E. No symptoms were observed on control pine needles treated with sterile water after 10 days; F–H. Symptoms on pine needles inoculated with conidial suspensions of DB 1-1, DB 1-2, and DB 1-3 after 10 days.

Five days after inoculation, needles inoculated with strains AH 1-1, AH 1-2, AH 1-3, AH 1-4, and AH 1-5 exhibited obvious gray spots (Fig. 6), while those inoculated with strains DB 1-1, DB 1-2, DB 1-3, DB 1-4, and DB 1-5 showed milder yellow–brown spots (Fig. 7). Ten days after inoculation, the lesions expanded into necrotic bands. The necrotic symptoms on needles inoculated with strains AH 1-1, AH 1-2, AH 1-3, AH 1-4, and AH 1-5 were pronounced, and the lesions had almost spread throughout the needles, forming necrotic bands with an incidence rate of 100%. The spots on needles inoculated with strains DB 1-1, DB 1-2, DB 1-3, DB 1-4, and DB 1-5 turned reddish brown and developed into necrotic bands, also with an incidence rate of 100%. No symptoms developed on the control needles. *Pestalotiopsis liuzhouensis* and *P. kendrickii* were successfully re-isolated from 100% of the inoculated plants, as identified by morphological characteristics and phylogenetic analysis of ITS, thereby fulfilling Koch’s postulates.

## ﻿Discussion

The species nomenclature of *Pestalotiopsis* was originally established and distinguished based on the first observation of their hosts (Jeewon et al. 2002; Lee et al. 2006). With the advancement of research, the genus has been classified using the morphological characteristics of conidia, such as the length and width of conidia, the length and color of intermediate cells, and the length and number of appendages, which have significantly improved the accuracy of species identification within the genus (Jeewon et al. 2003; Hu et al. 2007). Molecular technology has also been widely applied in fungal taxonomy and in the identification of plant fungal pathogens. *Pestalotiopsis* sensu lato was divided into three genera by Maharachchikumbura et al. (2014): *Pestalotiopsis* sensu stricto, *Neopestalotiopsis*, and *Pseudopestalotiopsis*. These three branches correspond to three conidial types—conidia with light brown or olivaceous concolorous median cells (*Pestalotiopsis* sensu stricto), conidia with versicolorous median cells (*Neopestalotiopsis*), and conidia with dark-colored concolorous median cells (*Pseudopestalotiopsis*). This classification further improves the accuracy of species identification and benefits studies on the species diversity of the genus (Liu et al. 2010). However, with the increasing number of new *Pestalotiopsis* species, additional challenges have emerged, such as conidial overlap and complexity and the absence of gene fragments in some species (Jeewon et al. 2002; Tejesvi et al. 2009). The separation of *Pestalotiopsis* species therefore remains a major challenge.

*Pestalotiopsis* is widely distributed worldwide and has numerous plant hosts (Maharachchikumbura et al. 2014). It has been reported and studied as a plant pathogen, saprophyte, and endophyte (Barr 1975; Maharachchikumbura et al. 2011). As a plant pathogen, *Pestalotiopsis* can cause a variety of diseases, including branch blight, leaf spots, canker, root rot, leaf chlorosis, branch withering, and fruit rot, and in severe cases can even lead to plant death, resulting in significant economic and ecological losses (Crous et al. 2011; Maharachchikumbura et al. 2014). Zhang et al. (2012) found that *Pestalotiopsis camelliae* can infect *Camellia japonica*, causing leaf withering; Karaca and Erper (2001) found that *Pestalotiopsis guepinii* can infect the branches of *Corylus heterophylla* and *Juglans regia*, causing the branches to turn brown and wither. Espinoza et al. (2008) found that *Pestalotiopsis clavispora*, *P. neglecta*, and *P. angustata* are pathogenic to the fruits of apple, kiwifruit, and blueberry, and that *P. clavispora* is also pathogenic to blueberry twigs. Li et al. (2024) found that *P. jiangsuensis* can infect *P. massoniana*, causing pine foliage to turn gray and wither. As an endophyte, *Pestalotiopsis* is typically nonpathogenic or weakly pathogenic to plant hosts (Watanabe et al. 2010).

Several reported endophytic *Pestalotiopsis* species can produce secondary metabolites with great potential applications in medicine, agriculture, and industry (Xu et al. 2010; Xu et al. 2014). For example, Strobel et al. (1996) isolated a strain of *P. microspora* from the bark of *Taxus chinensis*, which produces paclitaxel, an anticancer drug. *Pestalotiopsis microspora* has also been shown to produce another secondary metabolite with antifungal and antioxidant activities (Strobel et al. 2002). Shimada et al. (2001) isolated two plant growth regulators from the culture filtrate of *P. theae*. Yang and Li (2013) isolated the endophyte *P. foedan* from *Nelumbo nucifera*, whose fermentation broth produces a compound with moderate activity against tumor cell lines. Species of *Pestalotiopsis* are recognized as a rich source of diverse bioactive compounds and continue to show great potential for future development (Monden et al. 2013).

Interestingly, the pathogen causing needle blight of *P. massoniana* is not static. In 1980, Qiu et al. (1980) first discovered that the pathogen of *P. massoniana* needle blight was *P. funerea*, but Li et al. (2024) later identified another pathogen responsible for the same disease. Currently, two additional pathogens have been identified, indicating that pathogens of the same genus can exhibit diversity even on the same host. We suspect that the pathogen responsible for pinprick blight of *P. massoniana* may vary across regions, possibly due to geographical and environmental factors. Future research should expand the scope of investigation to better understand the relationships among these pathogens.

## Supplementary Material

XML Treatment for Pestalotiopsis liuzhouensis

XML Treatment for Pestalotiopsis kendrickii

## ﻿Additional information

### Use of AI

No use of AI was reported.

### Author ORCIDs

Hui Li https://orcid.org/0000-0002-2955-3106

De-Wei Li https://orcid.org/0000-0002-6035-1692

### Data availability

All of the data that support the findings of this study are available in the main text.

## References

[B1] Barr ME (1975) The genus *Ostreichnion*. Mycotaxon 3: 81–88. 10.5962/p.413961

[B2] Bate-Smith EC Metcalfe CR (1957) Leucanthocyanins. 3. The nature and systematic distribution of tannin in dicotyledonous plants. The Journal of the Linnean Society. Botany 55(362): 669–705. 10.1111/j.1095-8339.1957.tb00030.x

[B3] Bloomberg WJ (1971) Diseases of Douglas-fir seedlings caused by *Fusarium oxysporum*. Phytopathology 61: 467–470. 10.1094/Phyto-61-467

[B4] Carbone I Kohn LM (1999) A method for designing primer sets for speciation studies in filamentous ascomycetes. Mycologia 91(3): 553–556. 10.2307/3761358

[B5] Crous PW Summerell BA Swart L (2011) Fungal pathogens of Proteaceae. Persoonia 27: 20–45. 10.3767/003158511X606239 22403475 PMC3251321

[B6] Espinoza JG Briceño EX Keith LM Latorre BA (2008) Canker and twig dieback of blueberry caused by *Pestalotiopsis* spp. and a *Truncatella* sp. in Chile. Plant Disease 92(10): 1407–1414. 10.1094/PDIS-92-10-1407 30769572

[B7] Fu LY (1989) A newly discovered disease of *Pinus massoniana* in this province. Hunan Forestry Science and Technology (04): 34–35.

[B8] Glass NL Donaldson GC (1995) Development of primer sets designed for use with the PCR to amplify conserved genes from filamentous ascomycetes. Applied and Environmental Microbiology 61(4): 1323–1330. 10.1002/bit.260460112 7747954 PMC167388

[B9] Griffiths DA Swart HJ (1974) Conidial structure in two species of *Pestalotiopsis*. Transactions of the British Mycological Society 62(2): 295. 10.1016/S0007-1536(74)80038-0

[B10] Guba EF (1961) Monograph of *Pestalotia* and *Monochaetia*. Harvard University Press, 342 pp.

[B11] Hall TA (1999) BioEdit: A user-friendly biological sequence alignment editor and analysis program for Windows 95/98/NT. Nucleic Acids Symposium Series 41(41): 95–98. 10.1021/bk-1999-0734.ch008

[B12] He L Zhao C Yan M Zhang LY Xia YZ (2009) Inhibition of P-glycoprotein function by procyanidine on blood-brain barrier. Phytotherapy Research: PTR 23(7): 933–937. 10.1002/ptr.2781 19172664

[B13] Hu HL Jeewon R Zhou DQ Zhou TX Hyde KD (2007) Phylogenetic diversity of endophytic *Pestalotiopsis* species in *Pinus armandii* and *Ribes* spp.: Evidence from rDNA and β-tubulin gene phylogenies. Fungal Diversity 24: 1–22.

[B14] Hu RR Liang J Xie X Zhang YJ Zhang XY (2020) Incidence of pine needle blight and its relationship with site factors of Japanese red pine forests in the Kunyushan Mountains, East China. Global Ecology and Conservation 22: e00922. 10.1016/j.gecco.2020.e00922

[B15] Huang GF (2018) The best method of comprehensive control of loblolly pine blight and defoliation by exploring suspected pine wood nematode disease. Flowers 12: 364–368.

[B16] Huang Q He JS (2000) Identification and biological characteristics of pathogen of *Pinus tabulaeformis* blight. Sichuan Linye Keji 21(3): 28–30. 10.16779/j.cnki.1003-5508.2000.03.008

[B17] Jeewon R Liew ECY Hyde KD (2002) Phylogenetic relationships of *Pestalotiopsis* and allied genera inferred from ribosomal DNA sequences and morphological characters. Molecular Phylogenetics and Evolution 25(3): 378–392. 10.1016/S1055-7903(02)00422-0 12450745

[B18] Jeewon R Liew ECY Simpson JA Hodgkiss IJ Hyde KD (2003) Phylogenetic significance of morphological characters in the taxonomy of *Pestalotiopsis* species. Molecular Phylogenetics and Evolution 27(3): 372–383. 10.1016/S1055-7903(03)00010-1 12742743

[B19] Kang B Liu SR Zhang GG Chang JG Wen YG Ma JM Hao WF (2006) Carbon accumulation and distribution in *Pinus massoniana* and *Cunninghamia lanceolata* mixed forest ecosystem in Daqingshan, Guangxi, China. Acta Ecologica Sinica 26: 1320–1327. 10.1016/s1872-2032(06)60024-3

[B20] Karaca GH Erper İ (2001) First report of *Pestalotiopsis guepinii* causing twig blight on hazelnut and walnut in Turkey. Plant Pathology 50: 415–415. 10.1046/j.1365-3059.2001.00580.x

[B21] Katoh K Standley DM (2013) MAFFT multiple sequence alignment software version 7: Improvements in performance and usability. Molecular Biology and Evolution 30(4): 772–780. 10.1093/molbev/mst010 23329690 PMC3603318

[B22] Lee S Crous PW Wingfield MJ (2006) *Pestalotioid* fungi from Restionaceae in the Cape Floral Kingdom. Studies in Mycology 55: 175–187. 10.3114/sim.55.1.175 18490978 PMC2104724

[B23] Li WH Jing Y Yang JX (1995) Study on pine Nneedle rust disease of *Pinus massoniana* in the Qinba Mountain area. Xibei Linxueyuan Xuebao (04): 21–26.

[B24] Li GQ Wu WX Lu LQ Chen BY Chen SF (2023) Characterization of *Pseudofusicoccum* species from diseased plantation-grown *Acacia mangium*, *Eucalyptus* spp., and *Pinus massoniana* in Southern China. Pathogens (Basel, Switzerland) 12(4): 574. 10.3390/pathogens12040574 37111460 PMC10142214

[B25] Li H Peng BY Xie JY Bai YQ Li DW Zhu LH (2024) *Pestalotiopsis jiangsuensis* sp. nov. causing needle blight on *Pinus massoniana* in China. Journal of Fungi (Basel, Switzerland) 10(3). 10.3390/jof10030230 PMC10970983 38535238

[B26] Liang QX Pan FY Li DX (2002) Regularity of outbreak and control techniques of *Pinus massoniana* cercospora needle blight. Journal of Zhejiang Forestry Science and Technology 4: 64–65+84. 10.3969/j.issn.1001-3776.2002.04.018

[B27] Liu AR Chen SC Wu SY Xu T Guo LD Jeewon R Wei JG (2010) Cultural studies coupled with DNA based sequence analyses and its implication on pigmentation as a phylogenetic marker in *Pestalotiopsis* taxonomy. Molecular Phylogenetics and Evolution 57(2): 528–535. 10.1016/j.ympev.2010.07.017 20692352

[B28] Liu QH Zhou ZC Wei YC Shen DY Feng ZP Hong SP (2015) Genome-wide identification of differentially expressed genes associated with the high yielding of oleoresin in secondary xylem of masson pine (*Pinus massoniana* Lamb.) by transcriptomic analysis. PLOS ONE 10(7): e0132624. 10.1371/journal.pone.0132624 PMC4500461 26167875

[B29] Lu JY Chen H Yang ZQ Sun S Luo QF Xie JK Tan JH (2022) Physiological and molecular mechanisms of the response of roots of *Pinus massoniana* Lamb. to low-temperature stress. Frontiers in Plant Science 13: 954324. 10.3389/fpls.2022.954324 PMC9554314 36247576

[B30] Luo X Yu C (2020) First report of damping-off disease caused by *Fusarium oxysporum* in *Pinus massoniana* in China. Plant Disease 127: 401–409. 10.1007/s41348-020-00303-3

[B31] Maharachchikumbura SSN Guo LD Chukeatirote E Ekachai C Bahkali AH Hyde KD (2011) *Pestalotiopsis*-morphology, phylogeny, biochemistry and diversity. Fungal Diversity 50: 167–187. 10.1007/s13225-011-0125-x

[B32] Maharachchikumbura SSN Hyde KD Groenewald JZ Xu J Crous PW (2014) *Pestalotiopsis* revisited. Studies in Mycology 79: 121–186. 10.1016/j.simyco.2014.09.005 25492988 PMC4255583

[B33] Monden Y Yamamoto S Yamakawa R Sunada A Asari S Makimura K Inoue Y (2013) First case of fungal keratitis caused by *Pestalotiopsis clavispora*. Clinical Ophthalmology (Auckland, N.Z.) 7: 2261–2264. 10.2147/opth.s48732 PMC3848927 24348013

[B34] Monteiro P Gonçalves MFM Pinto G Silva B Martín-García J Diez JJ Alves A (2022) Three novel species of fungi associated with pine species showing needle blight-like disease symptoms. European Journal of Plant Pathology 162: 183–202. 10.1007/s10658-021-02395-5

[B35] Nguyen LT Schmidt HA von Haeseler A Minh BQ (2015) IQ-TREE: A fast and effective stochastic algorithm for estimating maximum-likelihood phylogenies. Molecular Biology and Evolution 32(1): 268–274. 10.1093/molbev/msu300 25371430 PMC4271533

[B36] O’Donnell K Cigelnik E (1997) Two divergent intragenomic rDNA ITS2 types within a monophyletic lineage of the fungus *Fusarium* are nonorthologous. Molecular Phylogenetics and Evolution 7(1): 103–116. 10.1006/MPEV.1996.0376 9007025

[B37] Orlikowski LB Ptaszek M Warabieda W (2014) Occurrence and harmfulness of *Pestalotiopsis funerea* to ornamental coniferous plants. Progress in Plant Protection 54(1): 25–30. 10.14199/ppp-2014-005

[B38] Qiu DX Tan SB Wu JC (1980) Preliminary study on red blight of *Pinus massoniana*. Forest Science (03): 203–207.

[B39] Quaedvlieg W Binder M Groenewald JZ Summerell BA Carnegie AJ Burgess TI Crous PW (2014) Introducing the consolidated species concept to resolve species in the Teratosphaeriaceae. Persoonia 33: 1–400. 10.3767/003158514X681981 PMC4312929 25737591

[B40] Shimada A Takahashi I Kawano T Kimura Y (2001) Chloroisosulochrin, chloroisosulochrin dehydrate, and pestheic acid, plant growth regulators, produced by *Pestalotiopsis theae*. Journal of Biosciences 56b: 797–803. 10.1515/znb-2001-0813 [Z Naturforsch]

[B41] Steyaert RL (1949) Contribution à l’étude monographique de *Pestalotia* de Not. et *Monochaetia* Sacc. (*Truncatella* gen. nov. et *Pestalotiopsis* gen. nov.). Bulletin du Jardin botanique de l’État a Bruxelles 19(3): 285–347. 10.2307/3666710

[B42] Strobel GA Yang X Sears J Kramer R Sidhu RS Hess WM (1996) Taxol from *Pestalotiopsis microspora*, an endophytic fungus of *Taxus wallachiana*. Microbiology 142(Pt 2): 435–440. 10.1099/13500872-142-2-435 8932715

[B43] Strobel G Ford E Worapong J Harper JK Arif AM Grant DM Fung PCW Chau RMW (2002) Isopestacin, an isobenzofuranone from *Pestalotiopsis microspora*, possessing antifungal and antioxidant activities. Phytochemistry 60(2): 179–183. 10.1002/chin.200238224 12009322

[B44] Sutton BC (1980) The Coelomycetes. Fungi imperfecti with pycnidia, acervuli and stromata. Commonwealth Mycological Institute, Kew, Surrey, UK. 10.1016/s0007-1536(81)80170-2

[B45] Tejesvi MV Tamhankar SA Kini KR Rao VS Prakash HS (2009) Phylogenetic analysis of endophytic *Pestalotiopsis* species from ethnopharmaceutically important medicinal trees. Fungal Diversity 38: 167–183.

[B46] Tint H (1945) Studies in the Fusarium damping-off of conifers. I. The comparative virulence of certain Fusaria. Phytopathology 35: 421–439.

[B47] Watanabe K Motohashi K Ono Y (2010) Description of *Pestalotiopsis pallidotheae*: A new species from Japan. Mycoscience 51: 182–188. 10.1007/s10267-009-0025-z

[B48] White TJ Bruns T Lee S Taylor J (1990) Amplification and direct sequencing of fungal ribosomal RNA genes for phylogenetics. In: Innis MA (Ed.) PCR Protocols: a guide to methods and applications.Academic Press, New York, 315–322. 10.1016/B978-0-12-372180-8.50042-1

[B49] Wu SX Wu J Wang Y Qu YF He Y Wang JY Cheng JH Zhang LQ Cheng CH (2022) Discovery of entomopathogenic fungi across geographical regions in southern China on pine sawyer beetle *Monochamus alternatus* and implication for multi-pathogen vectoring potential of this beetle. Frontiers in Plant Science 13. 10.3389/fpls.2022.1061520 PMC9832029 36643293

[B50] Xu J Ebada SS Proksch P (2010) *Pestalotiopsis* a highly creative genus: Chemistry and bioactivity of secondary metabolites. Fungal Diversity 44(1): 15–31. 10.1007/s13225-010-0055-z

[B51] Xu J Yang X Lin Q (2014) Chemistry and biology of *Pestalotiopsis*-derived natural products. Fungal Diversity 66: 37–68. 10.1007/s13225-014-0288-3

[B52] Xu Y Ren HT Wang P Zhao HX Song YQ Yu QF Liu XF (2017) Pathogenic fungi of Pinus sylvestris var. mongolica red blight. Xibu Linye Kexue 46(01): 91–95. 10.16473/j.cnki.xblykx1972.2017.01.017

[B53] Yang XL Li ZZ (2013) New spiral γ-lactone enantiomers from the plant endophytic fungus *Pestalotiopsis foedan*. Molecules (Basel, Switzerland) 18(2): 2236–2242. 10.3390/molecules18022236 23434873 PMC6269859

[B54] Yang Z Xia H Tan J Feng Y Huang Y (2020) Selection of superior families of *Pinus massoniana* in southern China for large-diameter construction timber. Journal of Forestry Research 31(2): 475–484. 10.1007/s11676-018-0815-2

[B55] Zhang YM Maharachchikumbura SSN Wei JG McKenzie EHC Hyde KD (2012) *Pestalotiopsis camelliae*, a new species associated with grey blight of *Camellia japonica* in China. Sydowia 64(2): 335–344.

[B56] Zhuang WY (2001) Higher Fungi of Tropical China. Mycotaxon, Ltd., Ithaca, New York, USA.

